# Extracellular Interactors of the IGF System: Impact on Cancer Hallmarks and Therapeutic Approaches

**DOI:** 10.3390/ijms25115915

**Published:** 2024-05-29

**Authors:** Caterina Mancarella, Andrea Morrione, Katia Scotlandi

**Affiliations:** 1 Laboratory of Experimental Oncology, IRCCS Istituto Ortopedico Rizzoli, 40136 Bologna, Italy; 2 Sbarro Institute for Cancer Research and Molecular Medicine and Center for Biotechnology, Department of Biology, College of Science and Technology, Temple University, Philadelphia, PA 19122, USA; andrea.morrione@temple.edu

**Keywords:** IGF system, cancer, extracellular signals, tumor microenvironment, extracellular vesicles, RAGE, target therapy, PROTACs

## Abstract

Dysregulation of the insulin-like growth factor (IGF) system determines the onset of various pathological conditions, including cancer. Accordingly, therapeutic strategies have been developed to block this system in tumor cells, but the results of clinical trials have been disappointing. After decades of research in the field, it is safe to say that one of the major reasons underlying the poor efficacy of anti-IGF-targeting agents is derived from an underestimation of the molecular complexity of this axis. Genetic, transcriptional, post-transcriptional and functional interactors interfere with the activity of canonical components of this axis, supporting the need for combinatorial approaches to effectively block this system. In addition, cancer cells interface with a multiplicity of factors from the extracellular compartment, which strongly affect cell destiny. In this review, we will cover novel extracellular mechanisms contributing to IGF system dysregulation and the implications of such dangerous liaisons for cancer hallmarks and responses to known and new anti-IGF drugs. A deeper understanding of both the intracellular and extracellular microenvironments might provide new impetus to better decipher the complexity of the IGF axis in cancer and provide new clues for designing novel therapeutic approaches.

## 1. Introduction

The insulin-like growth factor (IGF) system is a dynamic network of receptors, ligands and extracellular binding proteins necessary for normal cell functions, and it includes four membrane receptors (IGF1R, IR, IRR, IGF2R), three ligands (IGF1, IGF2, and insulin), and six extracellular IGF-binding proteins (IGFBPs) [1,2]. It mediates endocrine, paracrine, and autocrine signals, spanning long- to short-term effects, i.e., from development, growth and differentiation to glucose uptake and absorption, proteins, and lipids metabolism [3,4]. The multiplicity of responses orchestrated by this axis supports the evidence that unbalanced IGF activity is associated with several pathologies, including diabetes, growth retardation, neurodegenerative diseases, obesity, osteoporosis, and cancer [4,5]. However, such a multiplicity of effects is not exclusively due to the action of canonical components of this axis but is rather due to a complex network of regulators and interactors, which potentiate or antagonize IGF expression and/or function. In cancer, mutations in the IGF genes are relatively uncommon, with very few exceptions [6,7]. More frequently, transcriptional, post-transcriptional or functional alterations induce unbalanced IGF activity in tumor cells more than genetic ones [8,9]. Aberrant expression of oncogenic transcription factors (Sp1, HMGA1, TP53, BRCA2, WT1, VHL) [10,11,12,13] or fusion gene products (TMPRSS2::ERG, EWS::WT1, EWS::FLI1, PAX3::FKHR) might alter IGF axis activity in cancer [14,15,16,17]. At post-transcriptional levels, microRNAs (miRs), long non-coding RNAs (lncRNAs) and RNA-binding proteins, such as the family of N6-methyladenosine (m6A) readers RNA-binding proteins IGF2BPs, regulate the stability of IGF2 and IGF1R in tumor cells [18,19,20]. As recently reviewed, the complexity of IGF actions also relies on multiple intracellular interactions with critical effectors [8,9]. These mechanisms include the altered localization of IGF components, i.e., translocation of the IGF1R into the nucleus, endoplasmic reticulum or Golgi apparatus, and alterations in IGF1R internalization [21,22,23,24,25,26]. Other interactors affect the protein stability and ubiquitination of IGF, including E3 ligases NEDD4, MDM2, c-Cbl, and Cullin7 [27,28,29,30,31]. Functional interactors of the IGF system act on the plasma membrane, modulating its activity, including the discoidin domain receptor 1 (DDR1) [32,33,34], the proteoglycan decorin [8,35,36,37], beta-integrin, epidermal growth factor receptor (EGFR) and ALK [8,9,38].

Stimuli from the extracellular compartment can also deeply influence the activity of the IGF system, including hormones as well as dietary factors [39]. In addition, tumor cells functionally crosstalk with the tumor microenvironment (TME), which is a complex and heterogeneous ecosystem of non-malignant cells, including tumor-associated macrophages (TAMs), cancer-associated fibroblasts (CAFs), T, B, NK, and endothelial cells [40]. All these cell types contribute to extracellular matrix production, adhesion, immune regulation, and cytokine production. In addition, tumor cells are exposed to soluble factors, including extracellular vesicles, as well as restrictive conditions, including hypoxia and cell stress [40]. The complexity of the IGF actions plays a significant role in limiting the antitumor efficacy of anti-IGF agents in clinical trials [8]. In this review, we will discuss the action of the IGF system in cancer, highlighting critical molecular interactions and focusing on how molecular effectors from the extracellular compartment may provide previously unappreciated targets for molecular intervention.

## 2. General Overview of the IGF System

Insulin-like growth factor 1 receptor (IGF1R) and insulin receptor (IR) are trans-membrane tyrosine kinase receptors, sharing a 57% sequence identity, which reaches 85% in the kinase domains. IGF1R and IR are hetero-tetramers, composed of two αβ dimers joined by disulfide bridges [41,42]. The α chains are responsible for ligand binding, while the β chains are implicated in signal transduction. IGF1R is expressed in most cell types and plays a critical role in regulating proliferation, growth, and anti-apoptosis during development. IR exists in two isoforms, IR-A and IR-B, generated by alternative splicing occurring at exon 11 of the *INSR* gene [43]. IR-A lacks 12-amino acids encoded by exon 11 and represents the “oncofetal” form, primarily expressed in prenatal life and tumor cells. IR-B exerts metabolic functions, and it is mostly expressed in adipose tissues, muscle, and liver. The concomitant expression of IGF1R and IR-A/IR-B isoforms on cell membranes determines the stochastic formation of IR/IGF1R heterodimers called hybrid receptors (HRs). HRs, which include the combinations of IGF1R/IR-A and IGF1R/IR-B, are composed by one αβ monomer from the IGF1R and one monomer from the IR isoforms [44]. While the biological and structural properties of HRs are still not fully defined, recent evidence indicates that HRs functionally work in a manner more similar to IGF1R than IR, thereby promoting cell proliferation and anti-apoptosis more than glucose uptake [45]. On the contrary, Insulin-like growth factor 2 receptor (IGF2R), also known as the cation-independent mannose 6-phosphate receptor (M6P), is a type-1 transmembrane glycoprotein consisting of a large N-terminal extra-cytoplasmic domain and lacking intrinsic kinase activity [46]. IGF2R is generally considered a suppressor of IGF signaling by scavenging extracellular IGF2 [47]. However, it also interacts with other ligands, including M6P-labeled glycosylated proteins, leading to the transport of de novo synthesized proteins to lysosomes and playing a crucial role in cellular homeostasis [46]. The insulin receptor-related receptor (IRR) works as an extracellular pH sensor with a role in regulating acid-base balance in the kidneys. Functional studies reveal that mildly alkaline extracellular media activates the IRR, and its alkali-sensing machinery depends on multiple domains of its extracellular region [48].

The responses downstream from each receptor are connected to the ligand-binding affinities. In particular, IGF1 has the highest affinity for the IGF1R > IR/IGF1R hybrid > IR, IGF2 has the highest affinity for IGF1R/IGF2R > IR-A > IR/IGF1R hybrid >> IR-B, while insulin has the highest affinity for the IR >> IR/IGF1R hybrid > IGF1R [49]. 

There are six closely related high-affinity IGF-binding proteins (IGFBP-1 to IGFBP-6), which act in both IGF-dependent and IGF-independent manners [1].In the extracellular compartment, IGFBPs bind IGFs, but not insulin, and extend IGFs’ half-life from 10 to 30–90 min [1]. However, IGFBPs can also compete for the binding of ligands to cognate receptors, thereby inhibiting receptor activation [50]. IGFBPs modulate a network of functional interactions, which can further affect IGF-dependent responses. IGFBP-3 and IGFBP-5 can form ternary complexes with IGFs and the acid-labile subunit (ALS) glycoprotein (IGF/IGFBP-3/ALS), thereby extending the half-life of IGFs up to 20 h [1]. Other interactors include pregnancy-associated plasma protein-A (PAPP-A), a metzincin metalloproteinase that cleaves IGFBP-2, -4, and -5 in the extracellular compartment. PAPP-A activity determines the release of bioactive IGFs, thus enhancing IGF activity [51]. IGFBPs can also modulate IGF-independent functions by interacting with different non-IGF binding partners in the nucleus, cytoplasm, and plasma membrane, influencing cell survival, migration, DNA repair, and chromatin remodeling [52,53,54].

In the intracellular compartment, upon ligand binding, IGF1R, IR isoforms, and HRs undergo tyrosine transphosphorylation on the intracellular tyrosine kinase domain and subsequent recruitment of adaptor proteins, including the family of insulin receptor substrates (IRS) 1-6, Src homology 2 domain-containing transforming protein (Shc) and Grb proteins, particularly Grb2 and Grb10. Phosphorylation of these proteins determines the downstream activation of the phosphatidylinositol 3-kinase (PI3K)-AKT and the mitogen-activated protein kinases (MAPK) pathways [3,55,56]. In spite of the high homology between IGF1R and IR, recent phospho-proteomics studies have clearly demonstrated that the two receptors have distinct networks of downstream signaling components as, in fact, IR preferentially stimulates the AKT pathway, whereas the IGF1R preferentially stimulates MAPK pathway, indicating signaling specificities between the receptors [56].

## 3. The IGF System in Cancer: A Crucial Hub at the Crossroads between the Intracellular and Extracellular Compartments

Epidemiological, genetic, and experimental studies support the importance of the IGF system in tumorigenesis and cancer progression. Accordingly, an altered IGF axis has implications for various cancer hallmarks, including cell proliferation, protection from apoptosis, epithelial-to-mesenchymal transition (EMT) and metastases, vascularization, drug resistance, cell metabolism, epigenetic reprogramming, and DNA repair [8,9,57]. Previously published reviews have provided a detailed discussion of the mechanisms of IGF axis dysregulation in tumor cells [8,9]. With its set of transmembrane receptors, ligands and binding proteins, the IGF system stands at the intersection between the intracellular and extracellular compartments. Here, we focus on critical functional interactions occurring at this crossroads to explain the pleiotropic effects elicited by this axis in cancer [38].

One of these mechanisms involves the endocrine stimulus coming from the growth hormone (GH). GH is one of the main regulators of IGF1 production in the liver and other tissues and, accordingly, GH-IGF1 exerts tumor-promoting functions. Epidemiological evidence demonstrated that patients affected by Laron syndrome, a disease characterized by congenital IGF1 deficiency, due to the occurrence of homozygous mutations in the growth hormone receptor (GHR) or GH-induced intracellular signaling, do not develop cancer [58,59]. Laron syndrome patient-derived cells display the down-regulation of genes involved in the control of the cell cycle, motility, growth and differentiation (*CCNA1*, *CCND1*, *SERPINB2*, *AKT3*) and an up-regulation of metabolic genes involved in protection from oxidative and genotoxic insults (*UGT2B15*, *TXNIP*), as recently demonstrated by genome-wide profiling of Laron syndrome patients compared to normal controls [58]. On the other hand, high levels of IGF1 in the plasma have been positively associated with different tumor types, including colorectal cancer, breast cancer, prostate cancer, thyroid cancer and possibly malignant melanoma and multiple myeloma [60]. Different molecular mechanisms connect the GH/IGF1 axis to tumor progression. In colorectal cancer, IGF1 stimulation activates the IGF1R/AKT pathway and induces the transcriptional activation and expression of the transcription factor HOXA13, thereby promoting the in vitro migratory and invasive abilities of colorectal cancer cells and in vivo metastases formation [61]. IGF1 stimulation also induces a transcriptional program, which influences mitochondrial biogenesis and facilitates cancer progression [62,63]. Specifically, IGF1 stimulation induces the expression of PGC-1β and PRC proteins involved in the control of mitochondrial morphology and membrane potential [63]. In addition, IGF1 mediates a conserved signal through Nrf2 for the induction of BNIP3, regulating the synthesis and turnover of mitochondria in tumor cells [62]. IGF1 can also influence cancer cell metabolism. In B-cell precursor acute lymphoblastic leukemia (BCP-ALL), microarray analyses of BCP-ALL cells treated with IGF1 and IGFBP7, which binds and stabilizes the IGF1R on the surface and extends its response to IGF1 or insulin, promoted the up-regulation of genes involved in cell growth, including *PI3K/Akt/mTOR*, *mTORC1*, *MYC* targets, and metabolism, including oxidative phosphorylation-OXPHOS, adipogenesis, fatty acid metabolism, and glycolysis [64]. Sustained activation of the IGF1R/PI3K/AKT axis in BCP-ALL cells reduces GLUT1 recycling by blocking it at the cell surface, thereby enhancing glucose fueling and increasing glycolytic metabolism [64].

Cell–cell or cell–matrix adhesion processes can also alter the balance of the IGF system in tumor cells. Cadherin glycoproteins are adhesion molecules that play a critical role in cell–cell interactions. In cancer cells, the loss of E-cadherin is associated with enhanced anchorage independency and anoikis resistance. In addition, E-cadherin loss represents a crucial step during the EMT, a process profoundly influenced by the IGF axis. Indeed, the IGF pathway sustains the expression of transcription factors associated with the EMT, such as zinc finger E-box binding homeobox (ZEB)1 and ZEB2 [65]. In breast cancer models, the IGF1R regulates the expression and localization of YAP, a major mediator of the Hippo pathway, while YAP expression in turn up-regulates IGF1, a crucial mechanism underlying breast cancer stem cell progression [66]. Different findings demonstrate that E-cadherin negatively influences the activity of IGF1R [67]. Mechanistically, E-cadherin and IGF1R colocalize at the cell surface, an interaction disrupted by IGF1 stimulation [67]. In invasive breast ductal carcinoma and invasive lobular carcinoma, E-cadherin loss enhances the IGF1R action, invasion, and migration, which were further elevated in response to IGF1, serum, collagen I, and elevated AKT/MAPK signaling activation [68]. In this interaction, the loss of E-cadherin does not affect IGF1R levels, but it instead causes increased sensitivity to IGF1 [68].

In the cell–matrix interaction, decorin and lumican represent small leucine-rich proteoglycans and critical matrix constituents reported to modulate the functions of tyrosine kinase receptors in tumor cells, including IGF1R and IR-A. In vitro studies demonstrate that chondrosarcoma cells secrete lumican, an event supported by IGF1 action, suggesting the existence of a feedback loop [69]. From a functional standpoint, lumican promotes IGF1R activation at the cell membrane and activation of the MAPK intracellular signaling pathway, sustaining in vitro cell growth. In addition, lumican-deficient cells display increased expression levels of p53, suggesting that lumican might directly impact the resistance to the apoptosis of tumor cells [69].

On the contrary, different studies support the inhibitory role of decorin in responses evoked by the IGF axis [8,35,36,37]. Decorin binds with different affinities IGF1R, IR-A, IGF1, IGF2, and insulin, eliciting different inhibitory effects on these mediators. Interestingly, decorin inhibits IGF1-mediated phosphorylation of IGF1R, without affecting the IGF1R protein levels, thereby blocking the migratory and invasive capabilities of bladder cancer cells [36]. In addition, studies conducted on mouse embryonic fibroblasts homozygous for a targeted disruption of the *Igf1r* gene and stably transfected with the human IR-A isoform indicated that decorin significantly inhibited the IGF2-mediated cell proliferation of these cells by enhancing the IR-A protein degradation [35].

In the extracellular matrix, the integration of the IGF1R axis and integrin signaling strongly contributes to tumor progression. Integrins are cell surface proteins composed of alpha and beta subunit heterodimers primarily involved in cell–matrix interactions by binding substrates such as fibronectin. In cancer, integrins play a crucial role, particularly in cell migration. Overall, the functional interaction between IGF1R and integrins supports the oncogenic action of the IGF axis. IGF1R tyrosines (Tyr)1250/1251 phosphorylation is required for the formation of a complex containing IGF1R, β1 integrin and the scaffolding protein RACK1, a crucial event sustaining the turnover of focal adhesions and cancer cell migration [25]. Interestingly, adhesion-dependent IGF1R Tyr1250/1251 phosphorylation determines the rapid translocation of IGF1R from the plasma membrane to the Golgi apparatus, localization associated with the enhanced motility of tumor cells [25]. Recent evidence supports the relevance of the coupling of the IGF1R extracellular domain to a matrix adhesion receptor complex consisting of syndecan-1 (Sdc1) and αvβ3 or αvβ5 integrin for promoting cell growth and migration of neck cancer cells [70]. Accordingly, disruption of this complex using a Sdc1 peptide mimetic significantly reduced the in vitro and in vivo tumor cell survival and migration [70].

## 4. Novel Extracellular Interactors of the IGF System and Their Impact on Cancer Hallmarks

Cancer cells act in a complex tumor-specific microenvironment, which includes immune and stromal cells, nutrients, and extracellular vesicles, and shapes tumor cell behavior [38,40,71]. This section will highlight the critical extracellular interactors of the IGF system, defining the impact on tumor malignancy as well as the response to anti-IGF therapies.

### 4.1. The IGF System and Cancer-Associated Fibroblasts

Within the TME, CAFs represent non-malignant cells that provide support by producing extracellular matrix as well as various cytokines and growth factors. CAFs promote cancer progression through different mechanisms, including the secretion of growth factors. CAFs secrete high levels of the ligands IGF1 and IGF2, thereby promoting the tumor growth, migration, and invasion as well as drug resistance of IGF1R-expressing tumor cells [72,73,74]. Single-cell RNA-sequencing analysis of 50 primary gastric cancer samples has recently demonstrated the high heterogeneity of the gastric cancer TME, with at least four identified TME subtypes [74]. Of those, the subtype characterized by IGF1-overexpressing CAFs was specifically associated with chemo-resistance and gastric cancer recurrence. IGF1 secreted by CAFs induces drug-resistant phenotypes in gastric cancer cells through IGF1-α6β4 integrin ligand–receptor binding and activation of EMT biological processes [74]. In this context, characterization of the TME subtypes might identify a novel criterion for personalized treatment. On the contrary, other evidence demonstrates that cancer treatment might negatively affect CAFs by enhancing their secretion of IGF1/IGF2, with a negative impact on cancer progression. As shown in a rectal cancer model, preoperative radiotherapy to CAFs induces DNA damage, p53 activation and cell-cycle arrest [75]. Irradiated CAFs release IGF1, which mediates IGF1R/IR phosphorylation and activation of downstream signaling in tumor cells [75]. IGF1R signaling stimulates early increases in glucose uptake, lactate release, and subsequent changes in extracellular glutamine corresponding to enhanced transcription of genes implicated in glutamine metabolism and transport. These signaling events stimulate increases in cell number, spread, and in vivo multiorgan metastases [75]. Overall, this evidence indicates that chemoradiotherapy may indirectly elicit pro-survival signals through the stroma.

Paracrine signals from CAFs are also mediated by IGF2 as, in fact, exposure of colorectal cancer cells to CAF-derived IGF2 increases the activation of the IGF1R and downstream signaling, including the Hippo–YAP1 pathway [72]. For therapeutic intervention, colorectal cancer organoid and in vivo studies demonstrated the benefit of co-targeting IGF1R and YAP1 with the anti-IGF1R tyrosine kinase inhibitor (TKI) picropodophyllin (PPP) and verteporfin (VP), a YAP1 inhibitor [72]. CAFs exert pro-tumorigenic as well as anticytotoxic effects on breast cancer cells associated with the secretion of IGF2 [73]. CAF-derived IGF2 secretion activates IGF1R signaling in breast cancer cells by increasing the EMT as well as the in vitro and in vivo growth and migration. In addition, breast cancer cells in direct coculture with CAFs displayed resistance to doxorubicin treatment in 3D cell culture systems. The silencing of IGF1R or treatment with antibodies binding IGF1 and IGF2, BI836845 or xentuzumab inhibited the growth of in vivo cocultured CAFs-tumor cell xenografts [73].

In addition to IGF ligands, CAFs also secrete IGFBPs, with differential impacts on tumor cells’ drug sensitivity. Overall, while CAF-derived IGFs promote IGF1R signaling, CAF-derived IGFBPs might inhibit the IGF axis. As shown in gefitinib-resistant, EGFR-mutant PC9GR lung cancer cellular models, treatment with recombinant IGFBP induced drug sensitization, while the drug sensitivity was decreased by recombinant IGFs or conditioned media from CAFs in which IGFBP5 or IGFBP6 was depleted [76]. Thus, CAFs can either promote or reduce drug resistance, depending on the differential abundance of the secreted IGFs and IGFBPs. In the same study, the authors also found that the exposure of PC9GR-resistant cells to CAF-conditioned medium inhibited the compensatory FAK signaling activation induced by the EGFR inhibitor Osimertinib. Accordingly, co-targeting of IGF1R and FAK using small molecules recapitulated the CAF-mediated effects in culture and increased the antitumor efficacy of Osimertinib in mice [76]. Conversely, other reports demonstrate that treatment with the TKI dasatinib increased the expression of IGFBP6 in CAFs cocultured with chronic myeloid leukemia LAMA-84 cells and this was associated with an inflammatory state, TLR-4 signaling activation, and resistance to dasatinib [77]. Although the exact impact of this mechanism on IGF signaling still needs to be elucidated, the authors suggested the IGFBP-6 pathway as a potential candidate for therapeutic interventions to overcome resistance to TKI in the complex context of CAF-mediated effects.

### 4.2. The IGF System and Tumor-Associated Macrophages

TAMs represent crucial components of the TME, with reported tumor-promoting or tumor-suppressive functions. In general, TAMs hold the necessary plasticity to polarize toward M1-like phenotypes, with antitumor activity, or toward M2-like phenotypes, with tumor-promoting functions [78]. In terms of the crosstalk between TAMs and cancer cells, several reports indicate the capability of TAMs to secrete IGF1 and IGF2, similarly to CAFs. In breast cancer, systematic analysis by flow cytometry cell sorting of single-cell suspensions of tumor cells, non-immune stromal cells, and macrophages identified TAMs and CAFs as the main sources of IGF1/IGF2 ligands in the breast tumor microenvironment [79]. From a functional point of view, TAM-secreted ligands activate the IGF axis, leading to increased breast cancer cell proliferation and metastases. Accordingly, the authors found a statistical correlation between the activation of the IGF axis in cancer tissues, M2-like TAM infiltration and advanced tumor stage. A therapeutic combination of paclitaxel and the IGF1/2-neutralizing antibody xentuzumab significantly reduced the average metastatic lesion size and overall metastatic burden [79]. In thyroid cancer tissues, M2-like TAMs are highly enriched and in vitro studies demonstrate that they promote thyroid cancer cells’ invasion and stemness [78]. Cocultures between M2-like TAMs and thyroid cancer cells determined an increased invasive capability of tumor cells as well as increased sphere-forming capability and enhanced expression of stemness-associated markers such as CD133 and SOX2. From a mechanistic point of view, M2-like TAM-secreted IGF1/IGF2 activated the IR-A/IGF1R/AKT signaling in thyroid cancer cells, thereby sustaining the malignant phenotype. Inhibition of the AKT pathway using LY294002 significantly inhibited the invasion and stemness of the coculture system, supporting the connection between IGF signaling and TAM-mediated responses [78]. Similar results have been recently obtained in osteosarcoma, in which coculture experiments with TAMs and osteosarcoma cells indicate a critical role of TAM-produced IGF1 in osteosarcoma stemness [80]. In this study, the authors specifically demonstrated that TAM-secreted IGF1 acts on osteosarcoma cells and up-regulates RARRES2 expression, which maintains stemness through the NF-kB pathway and promotes chemotaxis of TAMs [80].

TAM-secreted IGFs can affect cancer cell behavior, while cancer cell-derived IGF can also have an impact on TAMs. An interesting body of evidence demonstrates that IGF1/IGF2 alters the TME by shaping macrophage polarization [81,82,83]. In hypopharyngeal squamous cell carcinoma, the crosstalk between cancer cells and TAMs mediates tumor progression through the transcription factor DACH1, which acts as a negative transcriptional regulator of IGF1 [82]. In this model, the loss of DACH1 is tumor cells enhances the secretion of IGF1, which in turn binds and activates the IGF1R and downstream signaling in TAMs. Coculture between TAMs and hypopharyngeal squamous cell carcinoma FaDu cells with short hairpin-mediated depletion of DACH1 displayed higher expression of phospho-JAK1 and phospho-STAT3 and increased expression of M2 macrophages markers (CD163, IL-4, IL-6, IL-10 and CD206) compared to TAMs cocultured with control cells. Functionally speaking, IGF1-polarized TAMs promoted in vitro migration and proliferation of cancer cells [82]. Other evidence in lung cancer supports the critical role of the endoplasmic reticulum (ER) chaperone protein GRP78 in the response to IGF1 stimulation in TAMs. As recently demonstrated, GRP78 is up-regulated in TAMs during M2 polarization, a condition associated with the in vitro and in vivo survival, proliferation, and migration of lung cancer cells [83]. The treatment of TAMs with IGF1 induces the translocation of GRP78 to the plasma membrane, favoring its association with IGF1R and subsequent activation of IGF1R/JAK/STAT downstream signaling and M2 polarization. In vitro data indicate the antitumor effects mediated by IGF1 blockade or GRP78 knockdown in TAMs, supporting the potential role of GRP78 as a therapeutic target in the complexity of the interaction between the IGF axis and TAMs [83].

### 4.3. The IGF System and T Lymphocytes

There is compelling evidence that the IGF axis in the TME promotes an anti-inflammatory, immunosuppressive response, enabling cancer cell expansion. The innate and adaptive immune systems surveil the TME and immuno-checkpoint molecules such as the programmed death protein 1 (PD-1) and its ligand PD-L1, B and T lymphocyte attenuator (BTLA), and cytotoxic T lymphocyte-associated protein 4 (CTLA4), and mediate inhibitory signals for naive T-cells, which become inactive and do not initiate antitumor immune responses in the presence of antigens [84]. Data from the literature support the notion that IGF down-regulation determines an immunogenic phenotype in tumor cells [85]. IGF1R targeting in breast cancer cells using small interfering RNA (siRNA) approaches not only decreased tumor growth in syngeneic mice but also triggered the features of an immune response [86]. Indeed, a delayed-type hypersensitivity assay increased strongly in mice immunized with IGF1R siRNA-transfected cells compared to control groups, suggesting that cellular immune responses were triggered by IGF1R down-regulation [86]. Other evidence indicated that the IGF1R inhibitor PPP affected autophagy and improved the therapeutic efficacy of PD-1 blockade in cancer-bearing mice, underscoring the capacity of the IGF1R antagonist to enhance chemoimmunotherapy efficacy in preclinical models [87]. This is reminiscent of a report from Ajona and colleagues, who demonstrated that the combined inhibition of IGF1R and PD-1 synergistically reduced tumor growth in mice injected with non-small-cell lung cancer (NSCLC) cells [85]. Interestingly, synergistical effects were achieved using both IGF1R inhibitors, short-term starvation or a fasting-mimicking diet, a condition that leads to reduced IGF1 plasma levels and inhibition of downstream signaling [85]. Notably, these data are based on the observation that caloric restriction is associated with increased immunosurveillance and favors antitumor CD8^+^ T-cell-mediated tumor cytotoxicity [88,89]. The use of anti-PD-1 monoclonal antibodies RMP1-14 in combination with short-term starvation significantly reduced tumor growth and increased survival compared to single treatments in 393P cells implanted in a syngeneic mouse model of KRAS-driven lung adenocarcinoma [85]. Similar results were obtained in a combinatorial treatment of starvation and anti-PD-L1 monoclonal antibody 10F.9G2 [85]. Overall, these data indicate that an active IGF1/IGF1R axis can counteract the therapeutic efficacy of anti-PD-1/PD-L1 agents. Accordingly, evidence obtained in 40 patients with NSCLC and treated with anti-PD-1/PD-L1 as monotherapy indicated that patients with clinical benefit (13 over 40) had significantly lower levels of circulating IGF1 and tissue IGF1R than patients who did not show clinical benefit [85]. The analysis of the infiltrate in tumors from mice treated with anti-PD-1 antibodies in combination with the IGF1R inhibitor PQ401 showed a significant increase of tumor-infiltrating CD8^+^, CD4^+^, natural killer and B cells, a decrease in intra-tumoral Treg cells, increased frequency of interferon-γ-producing T-cells and reduced expression of PD-1 and GITR in CD4^+^ and CD8^+^ T-cells. Collectively, this evidence indicates that combined treatment with the IGF1R inhibitor PQ401 and PD-1/PD-L1-blocking antibodies reverses the immune-suppressive microenvironment and promotes a specific antitumor immune response in lung cancer [85]. Other data demonstrate that insulin-induced PD-L1 expression in pancreatic ductal adenocarcinoma cells and coculture of pancreatic ductal adenocarcinoma cell lines and CD8^+^ T-cells indicated that insulin promoted tumor cell-mediated suppression of CD8^+^ T-cells’ proliferation, thereby conferring immune evasion [90]. This effect was partially dependent on IR-A/ERK/AP1 activation and subsequent AP1-mediated *PD-L1* promoter activation [90]. Notably, IR staining colocalized with PD-L1, underlining the association of the insulin/IGF axis with PD-L1-mediated immune control [90]. On the other hand, IGF1R and its signaling effectors are key downstream molecules of PD-L1 [91]. In addition to immune suppression of T-cells, PD-L1 elicits non-immune signaling in tongue cancer cells through the IGF/AKT axis. Accordingly, knockdown of PD-L1 determined decreases in both the PI3K/AKT and Raf/MEK/ERK pathways, inducing major changes in the EMT, proliferation, migration, invasion, and apoptosis [91].

**Figure 1:**
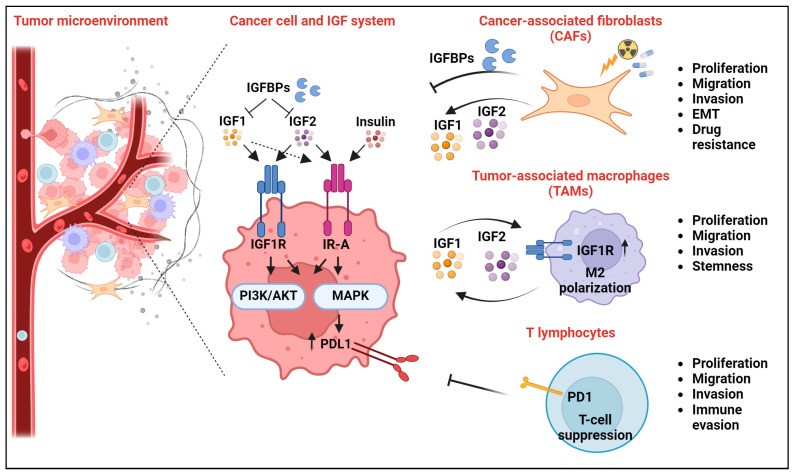
Schematic representation of the functional connections between the IGF system and major components of the tumor microenvironment (TME): cancer-associated fibroblasts (CAFs), tumor-associated macrophages (TAMs), and T lymphocytes. In cancer cells, IGF1 and IGF2 ligands bind with different affinities to IGF1R and IR-A, leading to the activation of the downstream PI3K/AKT and MAPK pathways. CAFs secrete IGF1, IGF2, and IGFBPs, eliciting the activation or inhibition of the IGF1R/IR-A axis, respectively, depending on their relative abundance. CAFs’ exposure to chemoradiotherapy enhances ligand secretion. TAMs secrete IGF1 and IGF2, leading to IGF1R/IR-A activation in cancer cells. Tumor-derived IGF1/IGF2 activate the IGF1R in TAMs (see the arrow), causing TAM polarization toward an M2-like pro-tumorigenic phenotype. The active IGF system in cancer cells favors the expression of the immune checkpoint inhibitor programmed death ligand 1 (PD-L1), which in turn binds to and inhibits the programmed death protein 1 (PD-1) on T-cells, causing T-cell suppression. Biological responses critical for cancer development and progression and functionally associated with the depicted interactions are reported on the right.

A schematic representation of the discussed molecular interactions between the IGF system and critical components of the TME, including CAFs, TAMs, and T-cells, in cancer is shown in Figure 1.

### 4.4. The IGF System and Extracellular Vesicles

Membranous structures derived from the endosomal system or the plasma membrane generate a heterogeneous population of vesicles which spans from exosomes to microvesicles [92]. Extracellular vesicles represent the major intercellular mediators of cell communication and work as cargos for different molecular species, including RNA, DNA, proteins, lipids, and non-coding RNAs [93]. In cancer, extracellular vesicles mediate both oncogenic or oncosuppressive functions, depending on their content and recipient cells, which can include both tumor cells as well as cells of the TME [92].

Reports from the literature indicate that tumor cells release in the extracellular compartment IGF1R incapsulated in extracellular vesicles, contributing to tumor progression, particularly metastases [94,95,96,97,98]. Extracellular vesicle analysis demonstrated that transmembrane IGF1R is present on the surface of extracellular vesicles as the unphosphorylated as well as phosphorylated active form [95]. In addition, DeRita and colleagues demonstrated that a variety of prostate cancer cell lines display enrichment of IGF1R, c-Src, and active Src^pY416^ in extracellular vesicles [94]. The authors suggested a model in which the crosstalk between IGF1R and Src leads to FAK activation and proliferation, metabolism, and protection from apoptosis. As discussed by the authors, this axis can be active both inside cells and inside extracellular vesicles and activated by extracellular vesicles in recipient surrounding cells [94].

A consistent body of evidence demonstrates that extracellular vesicles can transfer post-transcriptional regulators of the IGF axis, including miRs, lncRNAs, and RNA-binding proteins, thereby affecting IGF expression and functions in recipient cells with an impact on different hallmarks of cancer [99,100,101,102,103]. Of note, extracellular vesicles containing IGF component regulators can be produced either by cancer cells or by normal cells surrounding the tumor.

In glioblastoma, miR-603 regulated and inhibited the expression of IGF1R [102]. Interestingly, radiation exposure induced extracellular vesicle-mediated extrusion of miR-603 from the cancer cell, thereby de-repressing IGF1R. This IGF1R de-repression determined the increased expression of IGF1R, promoting a cancer stem cell state and radiation resistance in glioblastoma [102]. In non-small-cell lung cancer, extracellular vesicles from highly metastatic lung cancer cells promoted cell motility and the metastatic capabilities of recipient cells with a low metastatic potential through the cell-to-cell transmission of lncRNA MLETA1 [100]. From the mechanistic point of view, lnc-MLETA1 promoted cell migration by sponging miR-497-5p, thereby enabling the expression of IGF1R [100]. Other evidence in cancer cells demonstrates the role of RNA-binding proteins as regulators of the IGF system included in extracellular vesicles. Among them, the RNA-binding protein IGF2BP3, which sustains the translation of IGF1R in the cytoplasm of tumor cells, was encapsulated in extracellular vesicles extracted from Ewing sarcoma cell lines [20,103]. Interestingly, as we have recently demonstrated, extracellular vesicles derived from IGF2BP3-positive versus IGF2BP3-negative cells differentially influenced the phenotype of tumor recipient cells. Specifically, IGF2BP3-positive extracellular vesicles sustained the migration and invasive potential of recipient tumor cells as well as the expression of IGF1R and the downstream AKT pathway. This was partially attributed to a differential miRNA cargo of IGF2BP3-positive versus -negative cells as well as to the direct transfer of IGF2BP3 to the recipient cells [103]. Extracellular vesicles from normal cells can have an impact on the IGF axis activation and cancer cell behavior. For instance, miR99b-5p is down-regulated in prostate cancer tissue, while it is up-regulated in human bone marrow mesenchymal stem cells (HBMSCs) and targets IGF1R [101]. Interestingly, HBMSC-derived extracellular vesicles attenuated prostate cancer progression, leading to the inhibition of cancer cell proliferation, migration, invasion, and EMT, as assessed by the induction of E-cadherin in recipient tumor cells [101]. HBMSC-derived extracellular vesicles were able to transfer miR-99b-5p, thereby causing the suppression of IGF1R expression in recipient prostate cancer cells [101]. Other data demonstrate the critical role of extracellular vesicles derived from CAFs in the progression of esophageal squamous cell carcinoma. Specifically, CAF-derived extracellular vesicles promoted tumor growth in vivo in a xenograft mouse model since mice treated with CAF-derived vesicles displayed a higher tumor volume and increased lymph angiogenesis compared to mice treated with vesicles derived from normal fibroblasts [99]. Interestingly, this was attributed to a specific signature of microRNA in CAF-derived versus normal fibroblast-derived extracellular vesicles. Compared to normal fibroblasts, CAF-derived extracellular vesicles did not contain miR-100-5p, which targets IGF1R. Accordingly, vesicles from CAFs led to the activation of the IGF1R/PI3K/AKT pathway in cancer and endothelial cells, thereby accelerating lymph angiogenesis [99].

From the translational point of view, extracellular vesicle-associated IGF1R might represent a promising candidate for a circulating biomarker. Detection of IGF1R, along with other receptors, including EGFR and human epidermal growth factor receptor 2 (HER2), on extracellular vesicles has been recently confirmed using an immuno-PCR assay on samples from non-small-cell lung cancer patients, suggesting the feasibility of monitoring these tumor-associated membrane receptors in liquid biopsy [97]. In addition, an integrated database for exosome-based biomarker discovery (ExoBCD) evidenced IGF1R and FRS2 as the most promising prognostic circulating biomarkers for clinical use in breast cancer [98]. The recently developed Single Extracellular VEsicle Nanoscopy (SEVEN) assay from Saftics and colleagues showed that the evaluation of IGF1R successfully discriminated between extracellular vesicles derived from the plasma of pancreatic ductal adenocarcinoma patients with resectable disease and vesicles from plasma derived from healthy individuals [96]. Notably, the IGF1R-enriched extracellular vesicles from patients displayed a unique pancreatic cancer-enriched extracellular vesicle subpopulation based on the major features, including the dimensions and content of tetraspanin molecules [96].

**Figure 2:**
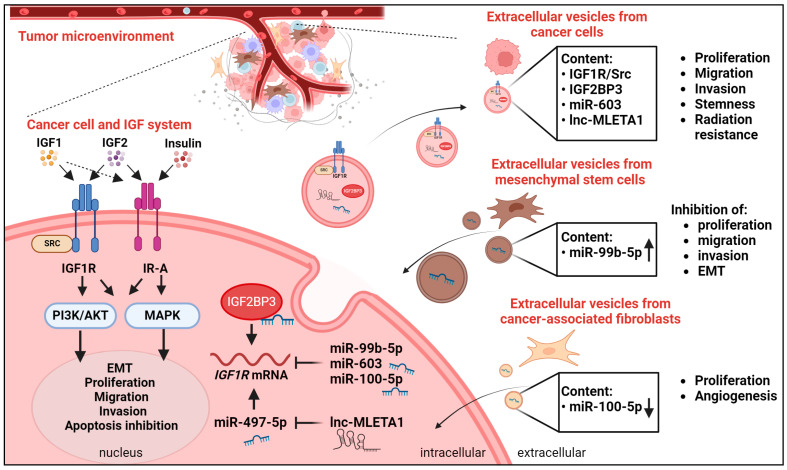
Schematic representation of the functional connections between the IGF system and extracellular vesicles in the tumor microenvironment (TME). In cancer cells, IGF1, IGF2, and insulin ligands bind with different affinities to IGF1R and IR-A, leading to the activation of the downstream PI3K/AKT and MAPK pathways and biological responses. IGF1R activates SRC to induce downstream signaling. At the post-transcriptional level, different regulators modulate IGF1R expression in the cytoplasm. The RNA-binding protein IGF2BP3 sustains IGF1R mRNA translation and expression. The depicted microRNAs (miRs) 99b-5p, 603, and 100-5p inhibit IGF1R expression. The long non-coding RNA (lncRNA) MLETA sponges miR-497-5p, thereby favoring IGF1R expression. Cancer cells as well as cells from the TME, including mesenchymal stem cells and cancer-associated fibroblasts (CAFs), secrete extracellular vesicles containing major interactors/regulators of the IGF system (up arrow indicates elevated content of reported microRNA while down arrow indicates low content of reported microRNA). Biological responses critical for cancer development and progression and functionally associated with the depicted interactions are reported on the right.

A schematic representation of the discussed molecular interactions between the IGF system and extracellular vesicles in cancer is shown in Figure 2.

### 4.5. The IGF System and Glycation

Hyperglycemia represents one of the major risk factors for cancer development. Accordingly, hyperglycemia is associated with cell proliferation, cell migration/invasion, apoptosis resistance, and resistance of tumor cells to chemotherapeutic drugs [104]. Multiple mechanisms underlie the effects of hyperglycemia on cancer cells [104]. For instance, hyperglycemia leads to the production of a wide range of pro-inflammatory factors, such as interleukin-6 (IL-6), tumor necrosis factor-α (TNF-α), and cyclooxygenase-2 (COX-2), all closely connected with pro-tumorigenic actions. Hyperglycemia can also increase the expression of matrix metalloproteinases (MMPs), favoring the hydrolysis of extracellular matrix components. Additional data demonstrate that hyperglycemia increases IGF1R signaling, but the exact mechanism is not fully elucidated. Notably, hyperglycemia represents one of the major adverse side effects linked to the use of anti-IGF system agents. One possible hypothesis is the fact that the drug-induced blockage of the IGF1R/IR induces insulin resistance [105]. Alternatively, the blockade of the IGF1R could alter the IGF1R/IGF1/GH axis. This can lead to the inhibition of the hypoglycemic effect of IGF1 or to elevated circulating levels of GH, causing an increase in liver glucogenesis and insulin resistance [105]. Recent findings support a connection between hyperglycemia, the IGF system, and glycation. In particular, hyperglycemia plays a pivotal role in the generation of glycated adducts called advanced glycation end products (AGEs). AGEs result from non-enzymatic glycation of proteins, nucleic acids, and lipids chronically exposed to elevated concentrations of glucose [106]. The Receptor for Advanced Glycation End Products (RAGE) is a transmembrane protein belonging to the immunoglobulin superfamily. RAGE is a relevant player in meta-inflammation and its signaling is aberrantly activated in conditions of dysregulation of the IGF/insulin axis, like obesity, type 2 diabetes, and cancer [107]. Initial studies in human monocytes demonstrated that AGEs induce the expression of IGF1 at a transcriptional level [108]. In vitro stimulation with the AGE protein increased IGF1 expression and secretion through RAGE [108]. On the contrary, other evidence showed that AGEs induced IGF1R transactivation and downstream activation of the AKT signaling pathway [109]. Mechanistically, RAGE couples to NAD(P)H oxidase to stimulate Src, which in turn phosphorylates and activates the IGF1R and downstream PI3K-AKT pathways [109]. Recent literature shows that the RAGE pathways may act as novel facilitators of IGF1 action in the protumorigenic crosstalk between cancer cells and the microenvironment. In breast cancer, the IGF1/IGF1R axis triggers STAT3-dependent transcriptional activation of S100A7, a cytokine-like molecule binding to RAGE [107]. S100A7 acts as a paracrine mediator in the breast tumor microenvironment, inducing the proliferation of human vascular endothelial cells and their assembly into vessel-like structures. The IGF1/IGF1R axis primes the breast tumor microenvironment toward the acquisition of an angiogenic phenotype through S100A7/RAGE signaling. A connection also exists between RAGE and IR as, in fact, recent findings indicated that RAGE and IR are co-expressed and associated with negative prognostic parameters in breast cancer. In situ proximity ligation assays and coimmunoprecipitation studies demonstrate that IR and RAGE directly interacted upon insulin stimulation and RAGE inhibition reduced cell proliferation, migration, and patient-derived mammosphere formation triggered by insulin. In vivo, the pharmacological inhibition of RAGE halted insulin-induced tumor growth, without affecting blood glucose homeostasis [107] and RAGE-induced insulin resistance, a condition associated with aberrant IGF activation [110]. Accordingly, in animal models of diet-induced obesity, RAGE deletion abrogated the establishment of insulin resistance [107]. Together, these findings suggest that targeting RAGE may represent a feasible therapeutic approach for blunting insulin-induced oncogenic signaling in breast cancer. Other recent data show that IGF1 and IGF1R expression correlated with AGEs in colorectal cancer patients who also had type 2 diabetes mellitus 111]. Overall, AGEs may influence the development of colorectal cancer in type 2 diabetes mellitus patients, supporting the hypothesis that it may be possible to lower the risk of colorectal cancer in the clinic by regulating AGEs through the modulation of blood glucose levels, which will then affect IGF-1 and its receptors [111]. A positive correlation between RAGE and IGF1 expression was also observed in ovarian serous carcinoma, suggesting that the RAGE and IGF1 levels may control the metastatic potential of this tumor [112].

**Figure 3:**
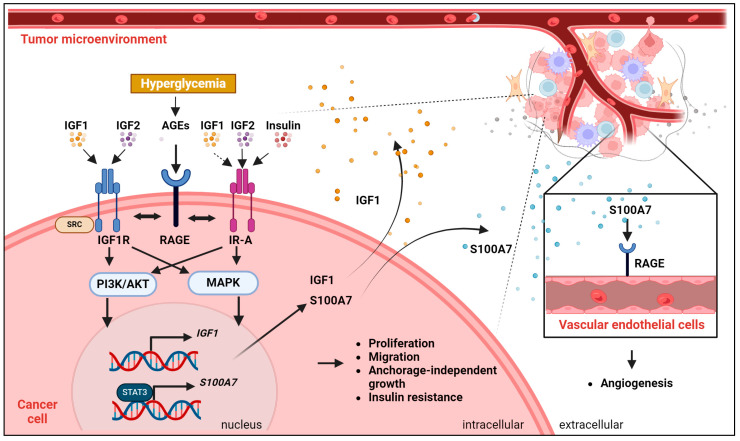
Schematic representation of the functional crosstalk between the IGF system and the Receptor for Advanced Glycation End Products (RAGE). In cancer cells, IGF1R and IR-A display high or low affinity for the ligands. Hyperglycemia favors the generation of AGE products, which activate RAGE. RAGE interacts with IGF1R and IR-A, modulating the activation of the PI3K/AKT/MAPK pathways and sustaining the transcription of IGF1 and the RAGE ligand S100A7. S100A7 additionally interacts with RAGE in vascular endothelial cells, sustaining angiogenesis. Biological responses critical for cancer development and progression and functionally associated with the depicted interactions are reported.

A schematic representation of the discussed molecular interactions between the IGF system and glycation in cancer is shown in Figure 3.

## 5. Emerging Therapeutic Strategies to Target the IGF System in Cancer

Given the preclinical and clinical evidence supporting a specific role for the IGF system in cancer onset and progression, different approaches have been tested in the last 25 years to inhibit this axis, which resulted in the development of three major classes of targeted therapies: monoclonal antibodies (mAbs) targeting the IGF1R, TKIs, and neutralizing antibodies targeting IGF ligands. Please see recent reviews for a wide discussion of the specific features and results of preclinical and clinical trials [5,38,113]. The great enthusiasm for these tools faced poor results obtained in clinical trials and the major issues linked to the use of these agents included a lack of antitumor efficacy, resistance mechanisms, and onset of adverse side effects, including insulin metabolism alterations. Of note, sarcomas, particularly Ewing sarcoma, represent a subgroup of tumor displaying an exceptional sensitivity to these agents, possibly due to the great biological dependency of these tumors on the IGF system, as we have recently reviewed [9]. However, most of the results from clinical trials, even in sarcomas, do not support further study of IGF axis inhibition as a single agent or administered in unselected cancer patients [114]. Table 1 summarizes a selection of relevant results available from clinical trials involving the major anti-IGF drugs.

**Figure 4:**
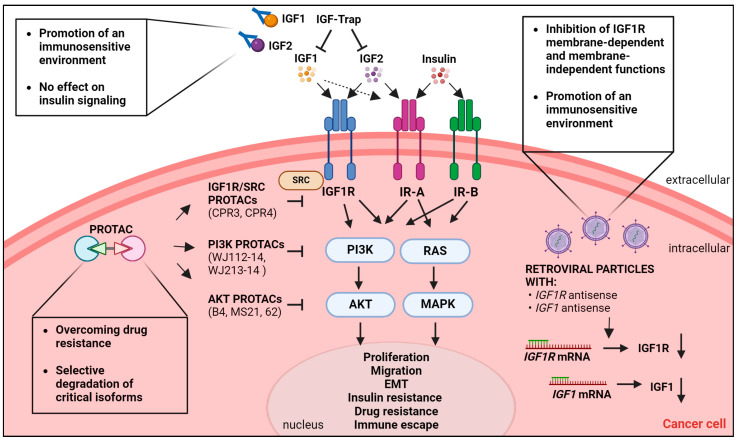
Schematic representation of emerging therapeutic strategies targeting the IGF system. In the extracellular compartment, IGF1, IGF2, and insulin bind their cognate receptors IGF1R, IR-A, and IR-B with different affinities. Receptor activation causes the activation of the downstream PI3K/AKT and RAS/MAPK pathways. Biological responses elicited by an active IGF system and critical for cancer development and progression are reported. The IGF-Trap binds to IGF1 and IGF2 but not insulin and blocks IGF1/IGF2 binding to the receptors. In the cytoplasm, different proteolysis-targeting chimeras (PROTACs) induce the degradation of various proteins of interest: 1. IGF1R and its interactor SRC; 2. PI3Kα and PI3Kβ isoforms of PI3K; 3. AKT. Transduction of retroviral particles containing IGF1R or IGF1 antisense oligos blocks IGF1R and IGF1 expression. The major advantages of the depicted therapeutic approaches are reported in boxes.

Alternative approaches have been developed in recent years, including IGF-Trap, gene therapy, and targeted-protein degradation-based strategies. In this section, we will focus on novel mechanistic evidence of new anti-IGF agents in an effort to highlight how these approaches might eliminate or attenuate the major limitations connected to the use of anti-IGF inhibition and the molecular complexity that surrounds the IGF system in cancer. A schematic representation of the discussed approaches in shown in Figure 4.

### 5.1. IGF-Trap

The IGF-Trap consists of the entire extracellular domain of IGF1R fused with the Fc domain of hIgG1. This IGF-Trap binds to IGF1 and IGF2 in the circulation but not insulin, thereby reducing their bioavailability and inhibiting activation of their cognate receptors and downstream responses [129,130,131,132]. The therapeutic efficacy of the IGF-Trap has recently been demonstrated in breast carcinoma, high-grade pediatric gliomas, triple-negative breast cancer, and pancreatic ductal adenocarcinoma using in vitro and in vivo models [129,130,131,132,133]. In addition, the IGF-Trap inhibited liver metastasis in mice injected with colon carcinoma MC-38 or lung carcinoma H-59 cells [130]. Overall, the IGF-Trap displayed potent inhibitory action on anchorage-dependent and -independent cancer cell growth and increased apoptosis. Combination studies evidenced synergistic effects between the IGF-Trap and fibroblast growth factor receptor (FGFR) inhibitors [132], while other data demonstrated that the IGF-Trap inhibited the growth of pancreatic ductal adenocarcinoma liver metastases by altering the tumor microenvironment [133]. In fact, the IGF-Trap reduced the recruitment and activity of several immunosuppressive cell types, thereby determining increased T-cell accumulation in the liver. IGF-Trap-treated mice displayed increased accumulation in the liver of CD4^+^ and CD8^+^ T-cells, a marked increase in CD11c+MHCII+ DC around metastatic foci. According to the immune profile induced by the IGF-Trap, the combination of the IGF-Trap and PD-1 inhibitors enhanced the in vitro and in vivo inhibitory effects on liver metastasis [133]. The results obtained with the IGF-Trap are very promising and in fact demonstrated better therapeutic profiles than anti-IGF1R mAbs and TKIs. In addition, the IGF-Trap displays low insulin affinity, thereby sparing toxicity associated with possible effects on IR signaling [130]. Accordingly, an ELISA assay performed on plasma from mice treated with the IGF-Trap indicated that the circulating insulin levels were not significantly different in treated mice compared to vehicle-treated control mice [130]. In addition, the IGF-Trap has high affinity for IGF2, thereby limiting IGF2/IR-A signaling, which represents a major resistance mechanism activated by tumor cells in response to anti-IGF mAbs or TKIs [134].

### 5.2. Gene Therapy

Inhibiting the synthesis of the IGF axis components represents another emerging approach for blocking this pathway in cancer. In this context, viral-based vectors represent a suitable approach for cancer gene therapy [135]. Genetically engineered retroviruses expressing IGF1R antisense RNA have been used to block IGF1R in cancer cells [136,137]. Samani and colleagues generated a replication-defective, vesicular stomatitis virus G-pseudotyped, Moloney murine leukemia virus retroviral vector in which an IGF1R antisense fragment was expressed as a bicistronic mRNA with an enhanced green fluorescent protein (EGFP) reporter under the control of a potent long terminal repeat (LTR), named the vLTR-IGF-1RAS retroparticle [137]. Transduction of these retroviral particles showed cancer gene therapy properties as, in fact, vLTR-IGF-IRAS retroviral particles reduced the IGF1R levels and inhibited the in vitro proliferation, migration, and invasion of highly metastatic carcinoma H-59 cells. In addition, vLTR-IGF-IRAS particles reduced hepatic metastases in mice generated after intrasplenic/portal injections of carcinoma cells. Recent evidence in glioma confirms the anti-cancer properties of vLTR-IGF1RAS particles, which determined a significant decrease in IGF1R expression, reduced cell proliferation, increased apoptosis, and reduced anchorage-independent growth in 3D spheroid assays [136]. In vivo experiments showed that mice with intra-cerebral implantation of vLTR-IGF-1RAS-transduced cells displayed longer survival, and developed smaller tumors, as compared to control-transduced group [136]. Targeting IGF1R expression could provide more effective therapeutic benefits in cancer compared to mAbs or TKI since this approach inhibits both the membrane-dependent as well as the membrane-independent functions of the receptor, including its ability to translocate into the nucleus or other intracellular organelles. In addition to targeting the IGF1R, evidence from the literature supports the use of anti-IGF1 gene antisense strategies targeting *IGF1* as an antitumor strategy [138,139]. In in vitro studies, transduction of IGF1 antisense RNA retroviruses inhibited glioma cells growth [139], while IGF1 AS-expressing episomal vectors have been used as anti-cancer vaccines [138]. Furthermore, in glioblastoma patients, IGF1 AS vaccines induced significant TCD8^+^ and TCD8^+^CD11b- immune responses as well as increased median survival [138].

### 5.3. Targeted Protein Degradation-Based Approaches

Targeted protein degradation represents an emerging cancer therapeutic strategy to control the protein levels of oncogenic drivers. Functionally speaking, this approach hijacks the intracellular ubiquitin–proteasome system to induce proteasome-mediated degradation of the proteins of interest [140]. Among these approaches, proteolysis-targeting chimeras (PROTACs) are heterobifunctional molecules composed of three modules: a ligand-binding domain for the protein of interest, a linker, and a ligand specific to the E3 ligase. PROTACs have already entered clinical trials and hold promise for overcoming the major limitations connected to standard targeted therapies, such as drug resistance, specificity or undruggable targets [140,141]. Specific PROTACs, named CPR3 and CPR4, have recently been designed to co-target the IGF1R and Src. This dual approach is based on preclinical evidence demonstrating the major role of Src activation in resistance to IGF1R mAbs or TKIs [142,143]. The CPR3 and CPR4 compounds induced the protein degradation of both the IGF1R and Src [143]. From a functional standpoint, the two compounds suppressed cell migration, invasion, and anchorage-dependent and -independent growth of breast and lung cancer cells, supporting the use of this strategy for inhibiting cancer progression [143]. Recently, novel PROTACs have been developed to target PI3K or AKT intracellular signaling molecules [144,145,146,147,148]. Among these novel PROTACs, the AKT degrader B4 induced over 95% AKT1 and AKT2 degradation in Jeko-1 cells, thereby reducing GSK-3β activation as well as impairing the proliferation of multiple cancer cells with a two-fold improvement compared to A0 [147]. The AKT degrader MS21 selectively degraded AKT and exerted superior in vitro and in vivo cell growth inhibition compared to the parent AKT inhibitor AZD5363 in mutant PI3K–PTEN pathway cell lines [145]. Notably, IGF1 or insulin enhanced the MS21 degradation of AKT in resistant KRAS- or BRAF-mutant cell lines, suggesting that the levels of AKT phosphorylation might influence PROTAC-mediated AKT degradation [145]. The novel AKT PROTAC 62 degrader derived from the AKT allosteric inhibitor ARQ-092 suppressed the proliferation of cancer cells harboring KRAS/BRAF mutations [144]. PROTAC 62 also degraded AKT in cancer cells harboring the PTEN/PI3K pathway mutation and was bioavailable in a mouse pharmacokinetic study via intraperitoneal administration. As recently published, WJ112-14 or WJ213-14 PROTACs display the ability to degrade specific isoforms of PI3K, PI3Kα and PI3Kβ, thereby avoiding total PI3K inhibition, which prompts insulin secretion, leading to metabolic adverse effects, including hyperglycemia and hyperinsulinemia [148].

## 6. Conclusions

Unbalanced IGF activity controls critical oncogenic processes, including cell proliferation, migration, EMT, glycolytic metabolism, and mitochondrial biogenesis. Multiple therapeutic agents, including mAbs, TKIs, as well as IGF-Trap, viral vectors, and PROTAC, have been developed and tested in preclinical and clinical settings. The multiplicity of effects elicited by this axis reflects the vast number of regulators and interactors that integrate in modulating IGF-dependent biological responses. The information obtained to date indicates that both intracellular and extracellular stimuli can either antagonize or potentiate the molecular signaling pathways mediated by this axis. A better understanding of these regulatory networks, coupled with advances in the medical chemistry of therapeutic strategies targeting the IGF system, might contribute to the identification of novel approaches to control cancer development and progression.

## Figures and Tables

**Table 1:** Selection of clinical studies involving anti-IGF agents in cancer.

Class of Therapy	Drug	Phase	Disease	Outcomes	References
Monoclonal antibodies	Robatumumab	II	Relapsed Ewing sarcoma and osteosarcoma	Osteosarcoma: 3/80 CR or PR; 23/80 SD Ewing sarcoma: 6/84 CR or PR; 23/84 SD	[115]
	Ganitumab (+ dasatinib)	I	Rhabdomyosarcoma	1/9 PR, 1/9 SD	[116]
	Ganitumab (+ palbociclib)	II	Relapsed Ewing sarcoma	2/10 SD	[117]
	Figitumumab (+ erlotinib)	III	Non adenocarcinoma non-small-cell lung cancer	16/293 PR, 113/293 SD	[118]
	Figitumumab	II	Squamous cell carcinoma of the head and neck	2/17 SD	[105]
	Cixutumumab (+ Temsirolimus)	I	Castration-resistant prostate cancer	3/16 SD	[119]
	Cixutumumab (+ capecitabine, lapatinib)	II	HER2-positive advanced breast cancer	No objective response	[120]
Tyrosine kinase inhibitors	Linsitinib (+ bortezomib and dexamethasone)	I	Relapsed/refractory multiple myeloma	No clinical benefit	[121]
	Linsitinib	II	Gastrointestinal stromal tumors	No objective responses	[122]
	AXL-1717	II	Non-small-cell lung cancer	24% CR + PR + SD	[123]
Neutralizing antibodies	Dusigitumab	I	Advanced solid tumors	13/39 SD	[124]
	Dusigitumab	I	Advanced solid tumors	4/10 SD	[125]
	Xentuzumab (+ enzalutamide)	Ib/II	Castration-resistant prostate cancer	No antitumor activity	[126]
	Xentuzumab	I	Advanced solid tumors	2/61 PR, 3/61 SD	[127]
	Xentuzumab (+ everolimus)	II	Breast cancer with non-visceral disease	No clinical benefit	[128]

CR, complete response; PR, partial response; SD, stable disease.
